# Three-dimensional soft tissue analysis of the hand: a novel method to investigate effects of acromegaly

**DOI:** 10.1007/s00238-016-1217-3

**Published:** 2016-07-12

**Authors:** Inge A. Hoevenaren, M. A. E. M. Wagenmakers, S. H. P. P. Roerink, R. T. Netea-Maier, D. J. O. Ulrich, Thomas J. J. Maal

**Affiliations:** 1 Department of Plastic, Reconstructive and Hand Surgery, Radboud University Nijmegen Medical Center, Geert Grooteplein Zuid 10, 6525 GA Nijmegen, The Netherlands; 2 Department of Internal Medicine, Division of Endocrinology, Radboud University Nijmegen Medical Center, Nijmegen, The Netherlands; 3 3D Lab of the Department of Oral and Maxillofacial Surgery, Radboud University Nijmegen Medical Center, Nijmegen, The Netherlands

**Keywords:** Acromegaly, Hand analysis, Three-dimensional imaging, Hand surgery, 3D stereophotogrammetry

## Abstract

**Background:**

Acral overgrowth is a highly common clinical sign in patients with active acromegaly. To what extent this overgrowth persists after long-term remission of acromegaly is largely unknown. Using the new imaging technique of three-dimensional (3D) stereophotogrammetry, it is possible to accurately investigate soft tissue changes of the hand. The aim of the recent study was to compare the 3D soft tissue characteristics of the hands of patients in long-term remission of acromegaly to those of a healthy pair matched control group.

**Methods:**

A case-control study was performed at a tertiary referral center. Twelve patients in remission of acromegaly (58 % male, mean age 58.3 years, mean BMI 29.6 kg/m^2^) were compared to twelve age-, gender-, ethnicity-, and BMI-matched control subjects. Of each individual, 3D photographs of both hands were acquired and analyzed using a 3D computer software program.

**Results:**

The patients in long-term remission of acromegaly have overgrowth of soft tissue of the hand compared to matched control subjects, with a larger length and width of the hand (*p* = 0.0025, *p* = 0.0017, respectively). Furthermore, the diameters measured at the proximal interphalangeal (PIP) joints of the individual fingers are larger in the acromegaly patients.

**Conclusions:**

Significant soft tissue overgrowth of the hand persists in former acromegaly patients, even after long-term remission. Analysis of 3D hand photographs is an accurate and easy tool to evaluate the acral soft tissue patterns in acromegaly.

Level of Evidence: Level III, diagnostic study.

## Introduction

Acromegaly is an uncommon clinical condition that is caused by prolonged exposure to immoderate quantities of growth hormone (GH). There is often a significant delay in diagnosis and treatment, since features of acromegaly develop insidiously [1]. Besides numerous metabolic changes, the GH excess causes proliferation of many tissues, including connective tissue, cartilage, bone, and skin [2], which causes musculoskeletal-related disorders and acral overgrowth. Musculoskeletal-related disorders account for the main functional disability in patients with acromegaly [3]. Following successful treatment of acromegaly, some features of the disease may show partial reversibility [4], but results are conflicting [5]. Concerning the hands of patients in long-term remission of acromegaly, the late effects of the disease have not been fully characterized [6]. Previous studies have focused mainly on calculations derived from radiographic images of the bony tissue of the hand [6–8]. None of these methods is a standardized method in the follow-up protocol so far. Furthermore, very little is known about the effect of acromegaly on soft tissue changes of the hand, which are equally affected [9]. This can be explained by the fact that until recently no reliable and proven effective method was available for analyzing soft tissue changes. In the past years, 3D imaging techniques have evolved rapidly and are increasingly used in clinical settings for soft and bony tissue imaging. Three-dimensional stereophotogrammetry has been developed for accurate soft tissue analysis. It is a fast technique that provides excellent geometry and texture information with good patient tolerance [10–13]. Furthermore, in contrast to frequently used imaging techniques like standardized radiographs, there is no use of harmful ionizing radiation. Therefore, 3D stereophotogrammetry provides a new opportunity to quantify acral disproportions in patients with acromegaly. Recently, we introduced a standardized method to analyze 3D stereophotographs of the hand [14].

The aim of the present study was to evaluate the differences in soft tissue characteristics of the hands between patients in long-term remission of acromegaly and matched control subjects. This study is the first study that uses 3D stereophotogrammetry of the hand in combination with the analysis according to Hoevenaren et al. to investigate the effects of a specific disease, namely acromegaly.

## Materials and methods

Adult patients in remission of acromegaly at least 2 years after successful pituitary surgery, visiting the Department of Internal Medicine, Division of Endocrinology, of the Radboud University Nijmegen Medical Center, were eligible for this case-control study. The diagnosis of acromegaly was based on clinical symptoms and biochemical tests, with remission being defined as disappearance of clinical signs of active GH hypersecretion and normalization of biochemical tests [9, 15]. Excluded were patients with a history of hand surgery and patients who received GH substitution. Twelve patients met all inclusion criteria and participated in this study. Seven patients were male, with a mean age of 58.3 years (SD 10.3) and a BMI of 29.6 kg/m^2^ (SD 4.3). Each patient was matched to a healthy age-, gender-, BMI-, and ethnicity-matched control subject, recruited via an announcement in a newspaper. They had no history of hand surgery or trauma and did not use hormonal substitutes. In the control group, seven subjects were male, with a mean age of 59.0 years (SD 10.5) and a BMI of 28.3 kg/m^2^ (SD 3.7). The age and BMI were pair matched between patients and controls.

**Fig. 1:**
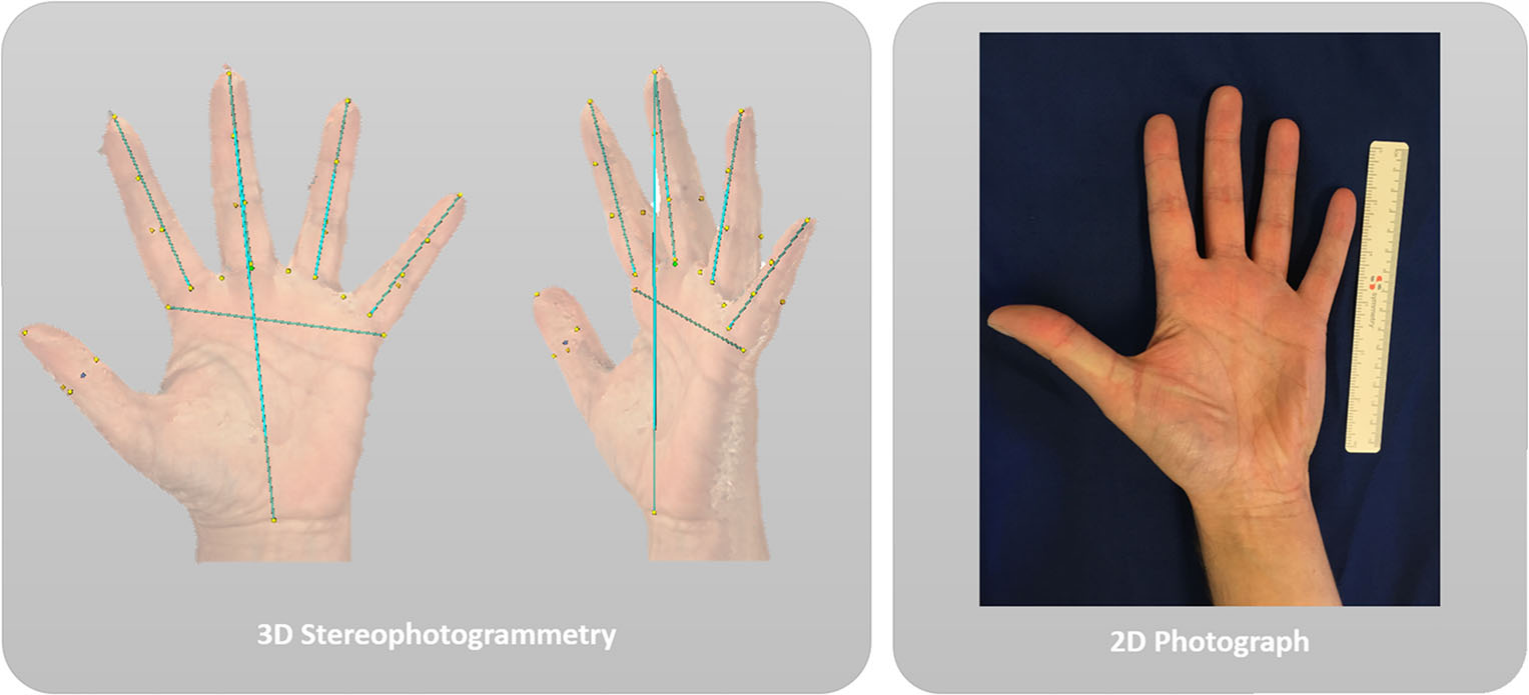
Example of 3D stereophotograph and 2D photograph of a control hand. An example is shown of a 3D stereophotograph of the hand (*left side*) and a normal 2D photograph (*right side*). In the photograph obtained through 3D stereophotogrammetry, markings and calculations can easily be done through the available software, as shown in the photograph by the *yellow dots* and *blue lines*

**Fig. 2:**
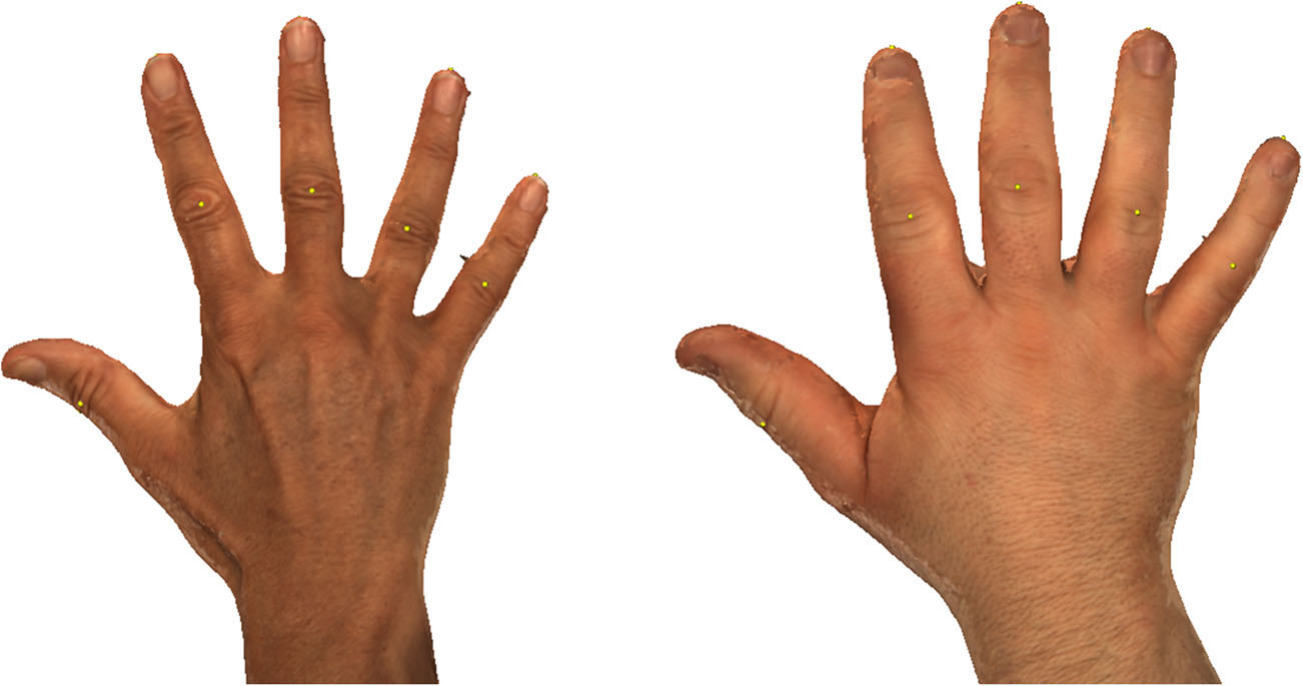
Example of 3D stereophotographs of an acromegaly and control hand. This figure shows an example of a 3D stereophotograph of the hand of an acromegaly patient (*right side*) and a matched control (*left side*). Clearly visible are the differences in finger and hand dimensions and the overall volume. The *yellow dots* are part of the landmark positioning

Three-dimensional stereophotographs were obtained of both hands from all patients and control subjects, using a stereophotogrammetrical camera set-up (3dMDCranial™ System, 3dMD LLC, Atlanta, USA). To exclude a recurrence of acromegaly, serum insulin-like growth factor type-1 (IGF-1) was determined in all patients on the day of the study. Figure 1 shows an example of an obtained stereophotograph of a control subject compared to a normal photograph, and Fig. 2 shows the 3D stereophotograph of a patient and a matched control. The photographs were taken by a trained technician following the principles described in the study by Hoevenaren et al. Using these principles, data from the 3D stereophotogrammetry were transferred to a 3D virtual model using Maxilim® software (Medicim NV, Mechelen, Belgium). A reference frame was set up according to a reproducible method, in order to align all hand models in the same orientation [14]. Thirty soft tissue landmarks were identified on each image (Table 1). After completing the landmark identification, measurements were automatically computed on each individual image using the different landmarks.

**Table 1:** Overview of landmarks

	Landmark	Abbreviation	Definition
1	Webspace 2	W2	The most inferior midpoint of the space between 2nd and 3rd finger
2	Webspace 3	W3	The most inferior midpoint of the space between 3rd and 4th finger
3	Webspace 4	W4	The most inferior midpoint of the space between 4th and 5th finger
4	DIP 2 midline	DIP2m	The midline of the 2nd DIP joint crease
5	DIP 3 midline	DIP3m	The midline of the 3rd DIP joint crease
6	DIP 4 midline	DIP4m	The midline of the 4th DIP joint crease
7	DIP 5 midline	DIP5m	The midline of the 5th DIP joint crease
8	MCP 2 midline	MCP2m	The midline of the 2nd MCP joint crease
9	MCP 3 midline	MCP3m	The midline of the 3rd MCP joint crease
10	MCP 4 midline	MCP4m	The midline of the 4th MCP joint crease
11	MCP 5 midline	MCP5m	The midline of the 5th MCP joint crease
12	MCP 2 radial side	MCP2r	The most radial point of the 2nd MCP joint crease
13	MCP 5 ulnar side	MCP5u	The most ulnar point of the 5th MCP joint crease
14	PIP 2 midline	PIP2m	The midline of the 2nd PIP joint crease
15	PIP 3 midline	PIP3m	The midline of the 3rd PIP joint crease
16	PIP 4 midline	PIP4m	The midline of the 4th PIP joint crease
17	PIP 5 midline	PIP5m	The midline of the 5th PIP joint crease
18	D1 top	D1t	The most distal midpoint of the fingertip of the 1st finger
19	D2 top	D2t	The most distal midpoint of the fingertip of the 2nd finger
20	D3 top	D3t	The most distal midpoint of the fingertip of the 3rd finger
21	D4 top	D4t	The most distal midpoint of the fingertip of the 4th finger
22	D5 top	D5t	The most distal midpoint of the fingertip of the 5th finger
23	Distal wrist crease (central)	DWC	The central point of the distal wrist crease
24	IP D1 radial side	IP1r	The most radial point of the IP joint crease
25	IP D1 ulnar side	IP1u	The most ulnar point of the IP joint crease
26	PIP 2 midline^a^	PIP2m	The midline of the 2nd PIP joint crease
27	PIP 3 midline^a^	PIP3m	The midline of the 3rd PIP joint crease
28	PIP 4 midline^a^	PIP4m	The midline of the 4th PIP joint crease
29	PIP 5 midline^a^	PIP5m	The midline of the 5th PIP joint crease
30	IP D1 radial side^a^	IP1r	The most radial point of the IP joint crease

Definitions of the 30 newly defined landmarks for the 3D photograph-based soft tissue analysis of the hand

^a^Landmarks on the dorsum of the hand

### Statistical analysis

The hand parameters were separately analyzed using the Holm’s method for correction for multiple testing [16]. The multiple correction was necessary for accurate comparison of the acromegaly patients with the control group. Per parameter a mixed linear model was fitted with fixed factor group (patient or control). To deal with the correlation of the data within a matched pair, the residual covariance matrix was not specified (unstructured).

Estimations of differences between patients and controls were done using the mixed linear model. The confidence intervals were corrected for multiple testing. Statistical significance was defined as *p* < 0.05.

## Results

Table 2 shows the calculated measurements derived from the defined landmarks. Hand width and length were significantly larger in the acromegaly patients compared to the healthy matched controls (*p* = 0.0017 and *p* = 0.0025, respectively). There was an average difference of 7.5 mm in the width and 9.1 mm in the length of the hands. The calculated diameter of the individual fingers at the level of the proximal interphalangeal (PIP) joint resulted in higher diameters in the patient group compared to the control group. In the third finger, this was a significant difference (*p* < 0.03); however, in the other fingers, there was a non-significant trend. These measurements indicate soft tissue overgrowth in the patient group. With respect to the length of the individual fingers, all fingers were larger in the acromegaly group; however, only the fifth finger showed a significant difference. Furthermore, we calculated the volumes of the hands of both groups. This resulted in a mean hand volume of 488.8 cm^3^ in the acromegaly group and 393.4 cm^3^ in the control group.

**Table 2:** Results

		Control		Patient		Patient vs control	
		Distance		Distance		Difference (95 % CI)	*p* value
		Left	Right	Left	Right		
Length of the hand	Mean	186.0	186.4	195.1	196.2	8.9	0.0025*
	SD	11.0	10.0	8.1	8.3	(3.0;14.9)	
Length of 2nd finger	Mean	73.9	74.5	76.4	76.4	2.3	0.2678
	SD	3.6	3.4	3.0	3.9	(−0.8;5.5)	
Length of 3rd finger	Mean	79.9	79.9	81.6	82.1	1.6	0.5429
	SD	4.5	3.9	4.0	4.4	(−1.5;4.8)	
Length of 4th finger	Mean	74.6	75.3	77.0	79.1	0.1	0.9335
	SD	4.0	4.2	3.3	4.8	(−2.1;2.2)	
Length of 5th finger	Mean	60.3	61.0	63.3	64.1	3.2	0.0132*
	SD	3.8	3.7	4.2	3.8	(0.6;5.8)	
Width of the hand	Mean	90.2	92.0	97.7	99.2	7.5	0.0017*
	SD	6.1	5.4	7.1	7.4	(2.7;12.4)	
Diameter at the 1st IP joint	Mean	13.4	13.8	14.4	15.7	0.5	0.5815
	SD	2.0	1.4	1.3	1.8	(−0.6;1.6)	
Diameter at the 2nd PIP joint	Mean	19.2	20.3	20.8	22.0	1.0	0.3033
	SD	1.9	1.5	1.6	1.7	(−0.5;2.6)	
Diameter at the 3rd PIP joint	Mean	20.2	20.7	21.6	22.4	1.7	0.0285*
	SD	2.2	1.7	1.7	1.7	(0.1;3.3)	
Diameter at the 4th PIP joint	Mean	18.8	19.6	20.9	20.9	1.8	0.0692
	SD	2.0	2.2	1.5	1.6	(−0.1;3.7)	
Diameter at the 5th PIP joint	Mean	16.7	17.0	17.9	18.7	1.0	0.4674
	SD	1.7	1.7	1.5	1.3	(−0.7;2.7)	

Results of the measurements of the control and patient group and calculated differences, including 95 % confidence interval (CI) between the patient and control group, with corresponding *p* values. Distances are in millimeters. Holm’s corrected *p* values are presented. Differences and CIs were calculated with the mixed linear model. Significant *p* values are appointed with an asterisk

## Discussion

This study is the first that uses 3D stereophotogrammetry of the hand in combination with specific 3D analysis to investigate the effects of a specific disease, namely acromegaly. To do so, we compared the 3D photographs of the hands of 12 patients in long-term remission of acromegaly and 12 healthy pair matched control subjects. Accurate digital models were created of the hands of every individual patient, which can be used in a clinical setting immediately. The digital models were analyzed using the recently published 3D soft tissue analysis according to Hoevenaren et al. In order to quantify soft tissue changes, we used different calculations on the predefined landmarks. Compared to the matched control group, the hands of the patients in long-term remission of acromegaly were 7.5 mm larger in width and 9.1 mm in length. This confirms the clinical impression that the hands are larger in patients in long-term remission of acromegaly. The length measurements of the individual fingers showed that only the fifth finger was significantly longer in patients in long-term remission of acromegaly, but there was a non-significant trend that all individual fingers were longer. This is an interesting finding, since in all our patients acromegaly was diagnosed during adulthood, after the closure of the epiphyseal growth plates.

Furthermore, the diameter at the proximal interphalangeal joints of the third finger was significantly larger, with a non-significant trend towards a larger interphalangeal joint in the other fingers. This could be, in combination with known persisting arthropathy [6] and the calculated volume difference, an explanation for the impaired joint function in patients in long-term remission of acromegaly, since a larger diameter in the 3D photographs is a sign of soft tissue overgrowth. A strength of this study is the matching of patients and control subjects for age, gender, ethnicity, and BMI. None of the previously published studies on problems of the hand in patients with acromegaly compared the patients to a matched control group. Furthermore, the 3D soft tissue analysis has a low intra- and interobserver measurement error, not exceeding 1 mm [17]. In this study, all measurements were performed by one experienced observer in order to reduce the magnitude of the measurement error even more. The relatively small sample size is a limitation of this study, which is caused by the strict inclusion and exclusion criteria and the fact that acromegaly is a rare disease. There are still certain disadvantages in the method and analysis described. As known from previous results, the landmark positioning in the first finger is less accurate than in other fingers and therefore we did not calculate the length of this individual finger. Additionally, there was no significant difference in the diameter measured at the interphalangeal joint of the first finger, although there was a non-significant trend. This might be explained by the small sample size and difficult landmark positioning. Recent improvements to the camera set-up are integrated in the photographing process, leading to more detailed photographs and more precise landmark positioning in future research. Another possible improvement for future research will be the integration of CT scan images of the hand to our current 3D photographs to characterize bony tissue [18]. This will lead to more detailed conclusions on what specific types of tissue are affected in patients with acral overgrowth.

### Clinical relevance and future perspectives

Our findings underscore once more that patients have to be informed that even after long-term remission of acromegaly the acral overgrowth persists. Besides esthetic concerns this may most likely lead to joint related symptoms and impaired movement, thus impairment in everyday activity and reduced quality of life [3]. Early stage counseling and hand therapy can be embedded as part of the treatment process and follow-up.

Furthermore, since the technique of 3D stereophotogrammetry of the hand is fast, accurate, relatively easy to perform, and harmless for the patient [10–12], it is a very promising technique for the follow-up of patients in both clinical and research settings. For acromegaly at present only blood values of IGF-1 and GH are recommended to detect possible disease activity and to evaluate the results of therapy or possible complications in the follow-up period [19]. However, especially during medical therapy, these values may not adequately reflect disease activity in peripheral tissues [20]. Using the 3D imaging technique, it is possible to compare different treatment options and their effect on soft tissues. Furthermore, the technique offers an additional patient-friendly method that could be easily used to investigate possible recurrences in an early state if laboratory measurements are conflicting. Although we have now demonstrated that acral overgrowth of the soft tissue of the hands is not completely reversible after long-term remission, it is still unknown whether it is at least partially reversible after remission and how long the process of remodeling takes. Longitudinal prospective studies are required to evaluate of what extent the acral overgrowth is reversible after remission and how different treatment modalities for acromegaly affect acral overgrowth.
